# Rapid quantification of underivatized amino acids in plasma by hydrophilic interaction liquid chromatography (HILIC) coupled with tandem mass-spectrometry

**DOI:** 10.1007/s10545-016-9935-z

**Published:** 2016-04-21

**Authors:** Hubertus C. M. T. Prinsen, B. G. M. Schiebergen-Bronkhorst, M. W. Roeleveld, J. J. M. Jans, M. G. M. de Sain-van der Velden, G. Visser, P. M. van Hasselt, N. M. Verhoeven-Duif

**Affiliations:** 1 Section Metabolic Diagnostics, Department of Genetics, University Medical Center (UMC) Utrecht, KC.02.069.1, Lundlaan 6, 3584 EA Utrecht, The Netherlands; 2 Department of Metabolic Diseases, University Medical Center (UMC) Utrecht, Utrecht, The Netherlands

## Abstract

**Background:**

Amino acidopathies are a class of inborn errors of metabolism (IEM) that can be diagnosed by analysis of amino acids (AA) in plasma. Current strategies for AA analysis include cation exchange HPLC with post-column ninhydrin derivatization, GC-MS, and LC-MS/MS-related methods. Major drawbacks of the current methods are time-consuming procedures, derivative problems, problems with retention, and MS-sensitivity. The use of hydrophilic interaction liquid chromatography (HILIC) columns is an ideal separation mode for hydrophilic compounds like AA. Here we report a HILIC-method for analysis of 36 underivatized AA in plasma to detect defects in AA metabolism that overcomes the major drawbacks of other methods.

**Methods:**

A rapid, sensitive, and specific method was developed for the analysis of AA in plasma without derivatization using HILIC coupled with tandem mass-spectrometry (Xevo TQ, Waters).

**Results:**

Excellent separation of 36 AA (24 quantitative/12 qualitative) in plasma was achieved on an Acquity BEH Amide column (2.1×100 mm, 1.7 μm) in a single MS run of 18 min. Plasma of patients with a known IEM in AA metabolism was analyzed and all patients were correctly identified.

**Conclusion:**

The reported method analyzes 36 AA in plasma within 18 min and provides baseline separation of isomeric AA such as leucine and isoleucine. No separation was obtained for isoleucine and allo-isoleucine. The method is applicable to study defects in AA metabolism in plasma.

**Electronic supplementary material:**

The online version of this article (doi:10.1007/s10545-016-9935-z) contains supplementary material, which is available to authorized users.

## Introduction

Amino acids (AA) are important metabolites for the human body. The metabolites are building blocks for peptides and proteins, can serve as sources of energy, are precursors for different metabolites such as neurotransmitters or can act as neurotransmitters themselves. Disturbances in AA concentrations are not only related to inborn errors of metabolism (IEM), but AA can also serve as sensitive markers for nutritional status. Besides, AA reflects the function of various organs (Duran, 2008). To diagnose amino acidopathies and for follow-up of therapy, AA analysis is commonly performed in biological specimens like plasma, dried blood spots (DBS), urine, and cerebrospinal fluid. The most commonly used strategies for AA analysis include cation exchange HPLC with post-column ninhydrin derivatization, GC-MS, and LC-MS/MS related methods (with and without derivatization). With respect to time, HPLC with post-column ninhydrin derivatization is less suitable for the modern laboratory with a requirement for a rapid turnaround time. Additionally, it is known that most of the derivatization methods used for LC-MS have major drawbacks, such as derivative instability and insufficient reproducibility of the derivative yield (Dolowy and Pyka 2014; Halket and Zaikin 2005; Kaspar et al 2009). With the introduction of ion-pairing agents, the separation of AA on a C18-reversed phase column improved. Complex derivatization procedures were no longer necessary and good separation of compounds was achieved (Piraud et al 2003; Piraud et al 2005). Unfortunately, problems with retention time stability and reduction of the sensitivity of the MS-detector in negative mode arose limiting the use of ion-pairing reagents (de Person et al 2008; Piraud et al 2005; Waterval et al 2009).

It is known that polar compounds such as AA and carbohydrates obtain good retention and separation by hydrophilic interaction liquid chromatography (HILIC) (Buszewski and Noga 2012; Greco and Letzel 2013; Kahsay et al 2014). Separation principle is based on the strong interaction of polar compounds with the hydrophilic phase. A benefit of HILIC is the use of an aqueous organic solvent eluent that enhances ionization. With the introduction of HILIC columns for UPLC new applications, e.g., for AA analysis became within reach. AA were analyzed semi-quantitative in fruit (Guo et al 2013; Zhou et al 2013), in rat brain (leucine) (Santos-Fandila et al 2014), and a subset of AA was analyzed in animal tissue hydrolysate (Krumpochova et al 2015). However, no such analysis was performed in human plasma using HILIC. The goal of this study was to develop a rapid, sensitive, and specific method for analysis and quantification of AA in human plasma without derivatization using HILIC tandem mass-spectrometry. The method had to be applicable to diagnose patients with inborn errors of amino acid metabolism. We validated this method according to ISO-15189 accreditation for medical laboratories and compared the results with those obtained using traditional ion-exchange chromatography.

## Materials and methods

### Reagents

2,3,3,3-D_4_-alanine, guanido-^15^N_2_-arginine:HCl, 2,3,3-D_3_-aspartic acid, 5-^13^C, 4,4,5,5-D_4_-citrulline, 2,4,4-D_3_-glutamic acid, 2,3,3,4,4-D_5_-glutamine, 1,2-^13^C_2_-glycine, U-^13^C_6_-histidine:HCl.H_2_O, U-^13^C_6_,U-D_10_,^15^N-isoleucine, 5,5,5-D_3_-leucine, 4,4,5,5,-D_4_-lysine:2HCl, methyl-D_3_-methionine, 5,5-D_2_-ornithine:HCl, ring-D_5_-phenylalanine, 2,3,3,4,4,5,5-D_7_-proline, 3-^13^C-serine, 1,2-^13^C-taurine, U-^13^C_4_, U-D_5_,^15^N-threonine, indole-D_5_-tryptophan, ring-D_4_-tyrosine, and 1-^13^C-valine were purchased from Cambridge Isotope Laboratories (Massachusetts, USA). D_9_-pipecolic acid was purchased from CDN-isotopes (Quebec, Canada). D_8_-homocystine was purchased from Buchem (Apeldoorn, the Netherlands), ^13^C_4_-^15^N_2_-asparagine, alanine, delta-aminolevulinic acid, arginine, asparagine, aspartic acid, argininosuccinic acid (ASA), citrulline, histidine, homocitrulline, homocysteïne, hydroxy-proline, isoleucine, leucine, lysine, methionine, methionine-sulfoxide, methionine-sulfone, phenylalanine, pipecolic acid, proline, serine, taurine, threonine, tryptophan, tyrosine, valine, ammoniumformiate, and DTT ((*2S*, *3S*)-1,4-bis(sulfanyl)butane-2,3-diol) were purchased from Sigma-Aldrich (Zwijndrecht, the Netherlands). Glutamic acid, glutamine, glycine, ornithine, sulfosalicylic acid (SSA), hydrochloric acid (HCl), and formic acid were purchased from VWR (Amsterdam, the Netherlands). Acetonitrile (ACN) and methanol both UPLC-grade were purchased from Biosolve (Valkenswaard, the Netherlands).

### Subjects

Blood samples were obtained for routine diagnostic purposes from patients suspected for an IEM and collected by venous puncture into heparin containing tubes (Greiner Bio-One, the Netherlands). For preparation of quality controls, blood was taken from a healthy volunteer. Blood was immediately centrifuged and stored at −20 °C. Blood samples were collected from (untreated) patients with a known inborn of AA metabolism including argininosuccinate lyase (ASL) deficiency (*n* = 2), 3-phosphoglycerate dehydrogenase deficiency (*n* = 4), citrullinemia type I (*n* = 1), hyperphenylalaninemia or phenylketonuria (*n* = 47), hyperprolinemia type I (*n* = 2), lysinuric protein intolerance (LPI) (*n* = 8), methionine adenosyltransferase (MAT) Ia deficiency (*n* = 3), maple syrup urine disease (MSUD) (*n* = 2), methylenetetrahydrofolate reductase (MTHFR) deficiency (*n* = 3), non-ketotic hyperglycinemia (NKH (*n* = 6)), ornithine aminotransferase (OAT) deficiency (*n* = 4), ornithine-transcarbamyolase (OTC) deficiency (*n* = 8), and tyrosinemia type I (*n* = 10). All IEM diagnoses in the control patients were confirmed by DNA-mutation analysis (data not shown). The samples obtained from the IEM patients were for routine diagnostics (clinical waste), analyzed anonymously, and not specifically collected for validation of this study.

### Preparation of calibrators

For the calibrators, a solution of 22 AA standards including hydroxy-proline (IS-I and ST-I, Table 1) was made, while for glutamine and asparagine a separate standard solution (IS-II and ST-II, Table 1) was prepared. The internal standards for IS-I were dissolved in 0.1 M HCl. Subsequently, to 8300 μL AA mixture (IS-I), 1700 μL 5 % SSA (final concentration 0.85 %) was added. From this 10 mL solution, 20 μL was added to plasma. IS-II was prepared by addition of 1600 μL labeled glutamine and 800 μL labeled asparagine to 7600 μL milliQ-water. From this 10 mL solution 20 μL was added to plasma. The final concentrations of the labeled AA (IS-I and IS-II) are shown in Table 1.

**Table 1:** Final concentrations for the internal standards (IS-I and IS-II, +MRM-transitions) and final concentrations for the calibrators ST-I and ST-II

(IS-I)	Amino acid	Concentration (μM)	MRM-transition (m/z)	(ST-I)	Amino acid	Concentration (μM)
1	2,3,3,3-D_4_-alanine	400	94.1 → 48.1	1	Alanine	2000
2	guanido-^15^N_2_-arginine	150	177.1 → 70.2	2	Arginine	500
3	2,3,3-D_3_-aspartic acid	150	137.0 → 75.1	3	Aspartic acid	200
4	5-^13^C, 4,4,5,5-D_4_-citrulline	750	181.2 → 75.1	4	Citrulline	200
5	2,4,4-D_3_-glutamic acid	400	151.1 → 87.0	5	Glutamic acid	500
6	1,2-^13^C_2_-glycine	600	78.0 → 31.1	6	Glycine	2000
7	U-^13^C_6_-histidine	200	162.1 → 115.1	7	Histidine	500
8	U-^13^C_6_,U-D_10_,^15^N-isoleucine	100	149.1 → 102.2	8	Isoleucine	500
9	5,5,5-D_3_-leucine	100	135.1 → 89.0	9	Leucine	1000
10	4,4,5,5,-D_4_-lysine	150	151.1 → 88.1	10	Lysine	1000
11	methyl-D_3_-methionine	50	153.0 → 107.1	11	Methionine	200
12	5,5-D_2_-ornithine	150	135.1 → 72.2	12	Ornithine	500
13	ring-D_5_-phenylalanine	3000	171.1 → 125.1	13	Phenylalanine	500
14	D_9_-pipecolic acid	25	139.1 → 93.1	14	Pipecolic acid	40
15	2,3,3,4,4,5,5-D_7_-proline	300	123.1 → 77.1	15	Proline	1000
16	3-^13^C-serine	400	107.0 → 60.9	16	Serine	1000
17	1,2-^13^C-taurine	150	128.0 → 110.0	17	Taurine	500
18	U-^13^C_4_,U-D_5_,^15^N-threonine	150	130.0 → 83.1	18	Threonine	1000
19	indole-D_5_-tryptophan	200	210.1 → 150.2	19	Tryptophan	500
20	ring-D_4_-tyrosine	100	186.1 → 140.1	20	Tyrosine	500
21	1-^13^C-valine	200	119.1 → 72.1	21	Valine	1000
				22	Hydroxy-proline	100
(IS-II)	Amino acid	Concentration (μM)	MRM-transition (m/z)	(ST-II)	Amino acid	Concentration (μM)
1	2,3,3,4,4-D_5_-glutamine	3200	152.1 → 88.1	1	Glutamine	2000
2	^13^C_4_-^15^N_2_-asparagine	400	139.0 → 77.0	2	Asparagine	400

IS-I and ST-I are prepared in 0.1 M HCl and IS-II and ST-II are prepared in milliQ-water

ST-I was prepared in 0.1 M HCl and ST-II was prepared in milliQ-water.

Standards IS-II and ST-II are unstable in 0.1 M HCl and need, therefore, to be prepared separately. After thawing of the standards, the calibrator mixtures were combined and were analyzed in a single injection. Calibrators were prepared in the range of 0, 20, 40, 60, 80, and 100 % of the calibration curve. The absolute concentrations of the calibrators are shown as supplementary data (Table 1). MilliQ-water was used as blank (0 %). All solutions were stored at −80 °C.

### Quality controls (QCs)

Three QC’s (QC-low, QC-middle, and QC-high) were prepared for quality control. AA concentrations were analyzed and the obtained values were in the middle range of the calibrators (QC-middle). This QC was subsequently diluted with physiological salt solution to obtain QC-low. For the preparation of QC-high AA, calibrator (ST-I and ST-II) was added to QC-middle. QC-low and QC-high were prepared to obtain AA concentrations in the range of approximately one-third and two-third of the calibration curve. The absolute concentrations of QC-low, QC-middle and QC-high are shown in the supplementary data (Table 2).

### Sample preparation

Forty μL plasma was mixed with 40 μL internal standard solution consisting of 20 μL labeled AA mixture (IS-I) and 20 μL labeled glutamine and asparagine (IS-II). After vortexing the patient sample and the IS-mixture, 280 μL solvent A (10 mM ammoniumformiate (final concentration) in 85 % acetonitrile + 0.15 % formic acid) was added. The sample was vortexed again, subsequently centrifuged for 5 min at 17,000×*g* in an Eppendorf centrifuge and all of the supernatant was transferred to a 96 wells-plate (Waters, Etten-Leur). After filtration with a 0.2 μM GHP-filter (VWR, Amsterdam) the sample was ready for analysis.

For the calibrators, 20 μL AA mixture (ST-I, consisting of 22 AA in 0.1 M HCl) was added to 20 μL (ST-II, dissolved in milliQ-water) (Table 1). To this 40 μL solution, 20 μL (IS-I) and 20 μL (IS-II) was added ending up with a total volume of 80 μL. The sample preparation for the standards is similar as described above.

Plasma samples with high AA concentrations were diluted until the concentrations of the AA were within the range of the calibration curve. A 50 fold dilution range was tested and acceptable and was set as a maximum. Plasma QC-samples (QC-low, QC-middle, QC-high) were analyzed within each series of samples. Argininosuccinic acid (ASA), ASA-anhydrides, hydroxy-proline, homocitrulline, homocysteine, homocysteine-cysteine-disulphide, homocystine, and the methionine oxidation products methionine-sulfoxide and methionine-sulfone were reported semi-quantitatively, while cystine, cysteine, and delta-aminolevulinic acid were reported qualitatively.

For method comparison, plasma samples were analyzed on the JEOL amino acid analyzer (Nieuw-Vennep, the Netherlands) (de Sain-van der Velden et al 2012; Duran 2008).

### Performance characteristics

Performance characteristics for ISO15189-accreditation for medical laboratories include accuracy, analytical sensitivity, analytical specificity, carry-over, clinical sensitivity, detection limit (LOD), interferences, limit of quantitation (LOQ), linearity, matrix effect, method comparison, repeatability, reproducibility, stability, and uncertainty of measurement.

The accuracy was determined by analyzing eight samples of the ERNDIM AA scheme. The analyzed samples included five samples from 2014 (AA181, AA182, AA183, AA184, and AA185) and three samples from 2015 (2015-01, 2015-02, and 2015-03).

Carry-over was determined by analyzing peak area after multiple injections. An average AA sample list consists of 35 injections including blanks, calibrators, QCs, and patient samples. An injection scheme is shown in the Table 3 of the supplementary data.

LOD and LOQ of each AA was determined based on the S/N-ratio for each AA in QC-low. LOD was calculated as three times signal to noise (S/N)-ratio, while LOQ was calculated as ten times signal to noise (S/N)-ratio. Obtained values were the mean of three independent experiments (performed at day 1, 5, and 10).

The matrix effect was estimated in triplicate for QC-low and QC-high and was determined as the ratio of the IS peak area after sample preparation and IS peak area in milliQ-water. In the Waters software tool Targetlynx, peak asymmetry parameters (measure at height 50 %, asymmetry windows 15 %) were set and data were evaluated. One hundred eight samples were reprocessed and expected asymmetry values (b/a) were calculated for every AA. The obtained asymmetry parameters were incorporated in the AA method for peak shape control. Additionally, absolute and relative intensities of the IS peak area were checked in every run in all patient samples.

Repeatability was determined by analysis of ten independent QCs (5x QC-low and 5x QC-high), that were analyzed within one run.

Reproducibility was determined by analysis of 20 independent QCs (10x QC-low and 10x QC-high). Each set of QCs (1x QC-low and 1x QC-high) was analyzed at ten consecutive days. The uncertainty of measurement was defined as twice the reproducibility.

### Chromatographic separation of AA

The chromatographic separation for plasma was carried out on an Acquity UPLC BEH Amide column (2.1×100 mm, 1.7 μm particle size) including a Van Guard™ UPLC BEH Amide pre-column (2.1×5 mm, 1.7 μm particle size) (Waters, Milford, USA). The column was maintained at a temperature of 35 °C and the sample volume injected was 2 μL. Optimal chromatographic separation was achieved at a flow-rate of 0.4 mL/min using a gradient with solvent A (10 mM ammoniumformiate in 85 % acetonitrile containing 0.15 % formic acid) and solvent B (10 mM ammoniumformiate in milliQ-water containing 0.15 % formic acid pH 3.0) as follows. Initial conditions were 100 % solvent A. After 6 min a gradient started for 0.1 min (6.0–6.1 min) and solvent A was decreased to 94.1 % and solvent B increased to 5.9 %. From 6.1 to 10 min solvent A was set at 82.4 % and solvent B was set at 17.6 % and from 10 to 12 min, solvent A was set at 70.6 % and solvent B was set at 29.4 % (Table 4, supplementary data). Then the column was equilibrated for 6 min in the initial conditions. Total run time was 18 min including column equilibration. The column was coupled to the mass spectrometer.

### Instruments

A Xevo-TQ MS triple quadrupole mass spectrometer with an electrospray ionization (ESI) source and an Acquity UPLC-system (Waters, Manchester, United Kingdom) were used. MassLynx software (v4.1; Waters, Manchester, United Kingdom) was used for instruments’ control and data acquisition. The mass spectrometer operated in ESI-positive mode, capillary voltage 1.00 kV, desolvation temperature 550 °C, source temperature 150 °C, cone gas flow 50 L/h, and desolvation gas flow was 1000 L/h. Collision energy and cone voltage were optimized for each AA and the compounds of interest were analyzed using multi reaction monitoring (MRM). The dwell time was set at automatically. The MRM-transitions for the AA internal standards and compounds of interest are shown in Tables 1 and 2, respectively.

**Table 2:** MRM-parameters and internal standards used for the analysis of amino acids

	Amino acid	RT (min)	MRM-transition (m/z)	Cone voltage (V)	Collision energy (V)	Internal standard
1	Tryptophan	2.6	205.1 → 146.0	18	18	indole-D_5_-tryptophan
2	Phenylalanine	2.6	166.1 → 120.1	20	15	ring-D_5_-phenylalanine
3	Leucine	2.9	132.1 → 86.1	15	10	5,5,5-D_3_-leucine
4	Isoleucine	3.1	132.1 → 86.1	15	10	U-^13^C_6_,U-D_10_,^15^N-isoleucine
5	Allo-isoleucine	3.1	132.1 → 86.1	15	10	–
6	Methionine	3.5	150.0 → 104.0	16	10	methyl-D_3_-methionine
7	Homocysteine	3.9	136.0 → 90.0	15	10	D_8_-homocystine
8	Homocysteine-cysteine disulphide	4.0	255.0 → 134.0	15	10	D_8_-homocystine
9	Valine	4.2	118.1 → 72.1	18	10	1-^13^C-valine
10	d-aminolevulinic acid	4.2	132.0 → 86.0	15	15	–
11	Proline	4.3	116.0 → 70.1	20	10	2,3,3,4,4,5,5-D_7_-proline
12	Tyrosine	4.4	182.1 → 136.1	20	15	ring-D_4_-tyrosine
13	Pipecolic acid	4.6	130.1 → 84.1	20	15	D_9_-pipecolic acid
14	Taurine	4.6	126.0 → 108.0	26	11	1,2-^13^C-taurine
15	Cysteine	4.9	122.0 → 76.0	12	10	–
16	Methionine-sulfone	5.1	182.1 → 56.0	20	18	methyl-D_3_-methionine
17	Alanine	7.0	90.1 → 44.1	15	8	2,3,3,3-D_4_-alanine
18	Hydroxy-proline	7.1	132.1 → 68.0	18	12	U-^13^C_4_,U-D_5_,^15^N-threonine
19	Threonine	7.7	120.0 → 74.1	15	10	U-^13^C_4_,U-D_5_,^15^N-threonine
20	Glycine	7.9	76.0 → 30.1	15	6	1,2-^13^C_2_-glycine
21	Methionine-sulfoxide	8.4	166.0 → 74.0	15	14	methyl-D_3_-methionine
22	Glutamine	8.6	147.1 → 84.1	15	15	2,3,3,4,4-D_5_-glutamine
23	Serine	8.6	106.0 → 60.0	15	10	3-^13^C-serine
24	Asparagine	8.7	133.0 → 74.0	16	14	^13^C_4_-^15^N_2_-asparagine
25	Homocitrulline	8.8	189.9 → 172.9	12	12	5-^13^C, 4,4,5,5-D_4_-citrulline
26	Citrulline	9.0	176.1 → 159.0	15	10	5-^13^C, 4,4,5,5-D_4_-citrulline
27	Glutamic acid	9.1	148.0 → 84.0	18	16	2,4,4-D_3_-glutamic acid
28	Aspartic acid	9.9	134.0 → 74.0	15	15	2,3,3-D_3_-aspartic acid
29	Histidine	10.3	156.1 → 110.0	15	15	U-^13^C_6_-histidine
30	Arginine	10.5	175.1 → 70.1	25	20	guanido-^15^N_2_-arginine
31	Lysine	10.8	147.1 → 84.1	20	15	4,4,5,5,-D_4_-lysine
32	Ornithine	10.9	133.1 → 70.1	15	15	5,5-D_2_-ornithine
33	Homocystine	10.9	269.0 → 136.0	15	10	D_8_-homocystine
34	ASA-anhydrides	11.0	273.2 → 70.2	25	25	5-^13^C, 4,4,5,5-D_4_-citrulline
35	Cystine	11.9	240.9 → 74.0	20	20	–
36	Argininosuccinic acid (ASA)	12.1	291.2 → 70.2	25	25	5-^13^C, 4,4,5,5-D_4_-citrulline

### Statistical analysis

Analyse-it (Microsoft, v2.12) was used for method comparison using Altman-Bland testing.

## Results

### Chromatographic separation of AA

All AA were baseline separated in less than 13 min including leucine and isoleucine. Total analytical run time was 18 min. The retention times of the individual AA are shown in Table 2.

### Validation

The performance characteristics of this method are shown in Table 3. Accuracy ranged from 78.8 % for asparagine to 121.8 % for aspartic acid.

**Table 3:** Performance characteristics (validation results) for the quantitative analysis of amino acids

	Amino acid	Accuracy (%)	Carry-over (%)	LOD (μM)	LOQ (μM)	Linearity (μM)	r^2^	Repeatability (CV %)	Reproducibility (CV %)	Uncertainty of measurement (CV %)
1	Tryptophan	101.7	0.00	0.0	0.0	0.0–250.0	0.999	2.7	3.5	7.0
2	Phenylalanine	98.0	0.03	0.0	0.0	0.0–250.0	0.998	2.3	3.9	7.8
3	Leucine	100.9	0.08	0.0	0.1	0.1–500.0	0.999	2.4	4.1	8.2
4	Isoleucine	108.2	0.03	0.1	0.3	0.3–250.0	0.998	2.5	5.3	10.6
5	Valine	93.8	0.04	0.1	0.4	0.4–500.0	0.994	6.0	5.9	11.8
6	Methionine	84.0	0.00	0.0	0.0	0.0–100.0	0.998	1.9	4.2	8.4
7	Proline	100.8	0.05	0.0	0.1	0.1–500.0	0.998	2.4	4.9	9.8
8	Tyrosine	100.6	0.12	0.0	0.1	0.1–250.0	0.998	3.8	4.0	8.0
9	Pipecolic acid	98.1	0.03	0.0	0.0	0.0–20.0	0.999	0.8	6.6	13.2
10	Taurine	93.0	0.15	0.0	0.0	0.0–250.0	0.993	2.5	4.7	9.4
11	Alanine	96.7	0.06	0.1	0.3	0.3–1000.0	0.999	1.9	3.5	7.0
12	Hydroxy-proline	98.8	0.00	0.0	0.0	0.0–50.0	0.997	3.0	16.7	33.4
13	Threonine	95.3	0.04	0.0	0.0	0.0–500.0	0.998	2.9	6.2	12.4
14	Glycine	97.3	0.03	0.0	0.1	0.1–1000.0	0.994	3.8	8.0	16.0
15	Glutamine	91.2	0.00	0.1	0.2	0.2–1000.0	0.996	1.8	5.2	10.4
16	Serine	84.6	0.33	0.0	0.1	0.1–500.0	0.994	4.8	5.4	10.8
17	Asparagine	78.8	0.00	0.0	0.0	0.0–200.0	0.995	3.0	6.2	13.2
18	Citrulline	94.6	0.02	0.0	0.0	0.0–100.0	0.998	4.0	5.3	10.6
19	Glutamic acid	114.2	0.00	0.0	0.1	0.1–250.0	0.998	3.4	4.4	8.8
20	Aspartic acid	121.8	0.74	0.0	0.0	0.0–100.0	0.993	6.9	10.8	21.6
21	Histidine	101.0	0.05	0.0	0.1	0.1–250.0	0.999	2.3	3.5	7.0
22	Arginine	108.6	0.09	0.0	0.1	0.1–250.0	0.998	1.7	4.6	9.2
23	Lysine	102.0	0.00	0.0	0.1	0.1–500.0	0.999	2.2	4.4	8.8
24	Ornithine	98.2	0.03	0.0	0.1	0.1–250.0	0.999	2.9	4.7	9.4

The r^2^ of the calibration curve was the mean r^2^ of the calibration curve measured at three independent days (day 1, 5, and 10)

During the validation experiments no relevant carry-over was noticed. In addition, no influence of interfering compounds such as clobazam, colecalciferol, depakine, diazepam, esomeprazol, levetiracetam, levocarnitine, macrogol, midazolam, ondansetron syrup, paracetamol, and thiamine or a special nutritional formula such as a ketogenic diet was noticed.

The LOD (range 0.0002–0.11 μmol/L) and LOQ (range 0.001–0.36 μmol/L) were calculated and were low enough to diagnose patients with an IEM of AA metabolism, thereby fulfilling the criteria of analytical sensitivity.

The matrix effect was studied and was highly variable for QC-low and QC-high ranging from 16.9 % for valine to 358.6 % for threonine respectively, but did not influence the LOD or LOQ.

The linearity of the calibration curve of each individual AA ranged from 20 μmol/L for pipecolic acid to at least 1000 μmol/L for alanine, glycine, and glutamine. Linearity was not further tested and for diagnostic purposes samples with AA concentrations above the highest value of the calibration curve were diluted with physiological salt solution and re-analyzed. The r^2^ of the calibration curve (Table 3) was the mean r^2^ of the calibration curve measured at three independent days (day 1, 5, and 10). Additionally, in Table 5 (supplementary data), the slope of each individual calibration curve measured at three independent days (day 1, 5, and 10) is presented.

The repeatability and reproducibility variations for the quantitative AA were determined by analyzing QC-samples. The repeatability (*n* = 5) for the quantitative AA tested ranged from 0.8 to 6.9 %, while the reproducibility (*n* = 10) ranged from 3.5 to 16.7 %. For hydroxy-proline, with the highest variation in reproducibility (16.7 %), labeled threonine was used for quantification instead of labeled hydroxy-proline. Therefore the analysis of hydroxy-proline can be considered to be semi-quantitative only. Reproducibility for all other AA was less than 10.9 %. The uncertainty of measurement was, with the exclusion of hydroxy-proline, below 21.6 % for all AA.

### Method comparison

As part of the validation procedure, the described method was compared with cation exchange HPLC with post-column ninhydrin derivation. Forty-one samples were analyzed using JEOL amino acid analyzer and with HILIC UPLC tandem mass-spectrometry. Six out of 23 AA including hydroxy-proline (3.7 μM, *p* < 0.01), serine (8.3 μM, *p* < 0.0001), aspartic acid (1.9 μM, *p* < 0.01), histidine (5.2 μM, *p* < 0.0001), lysine (9.0 μM, *p* < 0.0001), and ornithine (8.2 μM, *p* < 0.001) were significantly different. The small bias is of no clinical importance and therefore the differences were accepted.

Tryptophan is an AA that tends to bind to proteins and is sensitive to the deproteinization process. For the JEOL AA analyzer, AA are precipitated exclusively in 100 μL 5 % SSA and not in a combination of ACN and SSA. The use of SSA in particular is contra-indicated when quantifying tryptophan (Duran 2008) and therefore this AA was excluded for the comparison.

### Analysis of patient samples

To test the specificity of the method, plasma samples were analyzed from (untreated) patients with a known inborn error of AA metabolism (see Subjects section). In total, 97 plasma samples were analyzed and in 95 samples the metabolites of interest were elevated/reduced or identified, resulting in a clinical sensitivity of 97.9 %. In two plasma samples of MSUD-subjects elevated concentrations branched chain AA were identified. Since isoleucine and d-allo-isoleucine could not be separated, the patient samples were suggested to be of MSUD origin, but could not be definitely identified as MSUD.

In the plasma of two patients with MTHFR deficiency, the homocysteine-related metabolites were initially not detected, since homocysteine was lost during sample clean up. To overcome this problem, DTT (final concentration of 10.9 mmol/L) was added before the precipitation step (10 μM ammoniumformiate in 85 % acetonitrile + 0.15 % formic acid) and the samples were reanalyzed. Homocysteine was clearly present. In our laboratory, we have a method available for quantifying homocysteine (de Sain-van der Velden et al 2015) and since DTT was only added to qualify homocysteine in this method, we decided to leave the reducing agent out of the sample-precipitation step. Additional experiments showed that DTT can be safely added to the sample preparation procedure, without affecting the quantification of the compounds of interest (data not shown) and thus, quantification of homocysteine can be incorporated as well.

**Fig. 1:**
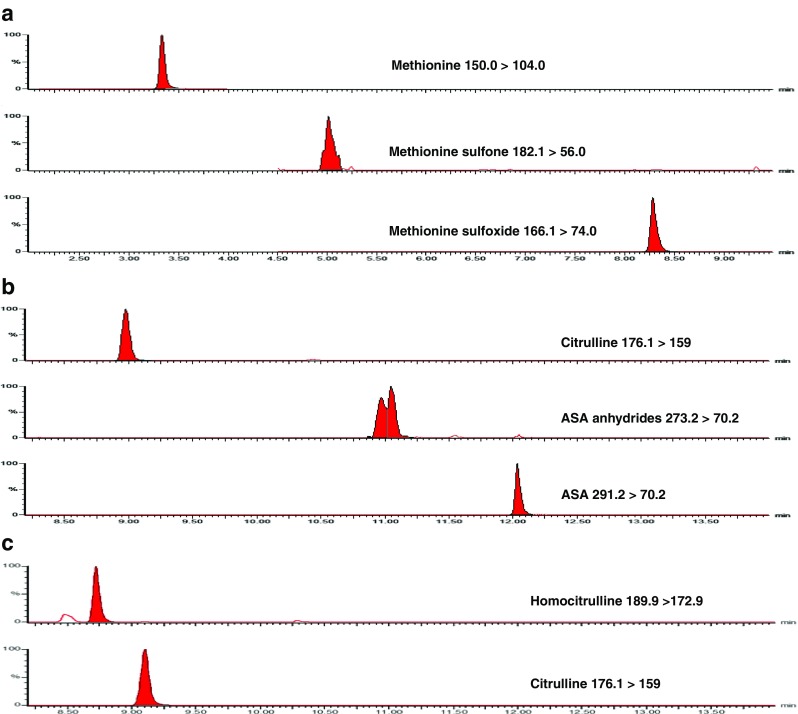
**a** Amino acid abnormalities observed in a plasma specimen of a patient with MAT-Ia deficiency, **b** Amino acid abnormalities in a plasma specimen of a patient with ASL-deficiency treated with citrulline, ASA: argininosuccinic acid, **c** Amino acid abnormalities in a plasma specimen of a patient with citrullinemia type I

In Fig. 1, the abnormal metabolites in a patient with MAT-Ia, ASL-deficiency on treatment and citrullinemia type I are shown. In MAT-Ia deficiency, a high methionine was observed. The sulfur-group of methionine can oxidize to form methionine-sulfoxide and methionine-sulfone (Fig. 1a). For diagnostic follow-up of the high methionine, the sample was re-analyzed after the addition of DTT (final concentration of 10.9 mmol/L) and a slightly elevated homocysteine was noticed (data not shown) that was quantified (14.5 μmol/L, *n* < 12.5 μmol/L) according to the method described in (de Sain-van der Velden et al 2015). In Fig. 1b, the AA abnormalities of a patient with ASL-deficiency on treatment (citrulline) was shown. As a consequence of the metabolic block, argininosuccinic acid (ASA) accumulates and loss of water results in the formation of ASA-anhydrides. In Fig. 1c, the AA abnormalities in a patient with citrullinemia type I was shown. Next to an increased concentration of citrulline, the presence of homocitrulline was noticed.

In approximately 30 min (including sample preparation) a total AA profile is generated. One can argue, that this is incorrect, since for quantitative purposes a calibration curve needs to be analyzed as well. Indeed, for every series of AA, we analyze a calibration curve consisting of six calibrators increasing the analysis time for urgent diagnostics with approximately 110 min. Therefore, we investigated whether it was possible to calculate AA in a plasma sample on a calibration curve that was analyzed earlier. Twenty-one samples in which AA were analyzed, were reprocessed and calculated using three different (independent) calibration curves that were analyzed during the previous week. The data were reproducible except for asparagine. The CV for the other AA was below 15 % and was lower than the uncertainty of measurement of the analysis. This implies that for urgent diagnostics a temporary interpretation of the AA results can be given within 30 min (including sample preparation and sample processing).

## Discussion

Here, we present an accurate and rapid method using HILIC-chromatography coupled to tandem mass-spectrometry for analysis of AA and related metabolites in human plasma. The method was validated according to ISO15189 accreditation for medical laboratories and can be used for diagnosis and follow-up of patients with an IEM of AA metabolism.

The method is fast, the sample preparation is simple, no derivatization procedure is needed, and the total runtime (including equilibration) is only 18 min. The method is sensitive (low LOD and LOQ) and reproducible. Currently, more than 6000 AA runs (approximately 1500 analyses/column) have been performed in our lab and no shift in retention times has been observed, implying that in contrast to other tandem mass-spectrometric methods (Piraud et al 2005; Waterval et al 2009) HILIC-chromatography is stable over time. From our experience, for assessing column performance, aspartic acid and tyrosine are the first 2 AA for which the peak shape worsens, while leucine and iso-leucine are still baseline resolved.

Recently, Krumpochova et al (Krumpochova et al 2015) compared different AA analysis procedures and reported that HILIC showed the least accurate results and the longest analysis time (40 min) in comparison with GC-MS and LC-MS. In the method reported here, total analysis time was 18 min and nearly a double amount of compounds are quantified. In contrast to the HILIC method described by Krumpochova et al (Krumpochova et al 2015) we used a different column, different mobile additives, and a lower pH-value for separation of AA. It is well known that a reduction in particle size could provide higher sensitivity and selectivity, a better resolution, narrower peaks, and shorter retention when analyses are performed on a 1.7 μm particles column instead of a 5 μm particles column. In addition, Guo et al (2013) showed that ammoniumformiate used as a salt additive of the mobile phase and a lower pH value of the buffer, resulted in much better peak shapes for AA analysis. With these alterations in mind, and since the method is easy to adapt, we believe that HILIC is favorable to GC- and LC-MS methods.

In the validation procedure, we tested the specificity of this method by analyzing AA profiles of patients with a known IEM of AA metabolism. In plasma specimens of two MSUD-patients (classical form), in which an increased concentration of D-allo-isoleucine was initially demonstrated by JEOL amino acid analyzer, D-allo-isoleucine was not identified. As is known, D-allo-isoleucine can be normal in MSUD (Puckett et al 2010), but this was not the case in these two patients. D-allo-isoleucine and isoleucine have an equal molecular weight and D-allo-isoleucine was not separated from isoleucine. Therefore, we investigated an alternative approach for diagnosing MSUD. The region4database is for NBS samples analyzed from bloodspots (McHugh et al 2011) and data from this region4database show that the median for leucine is higher than valine in patients with MSUD. Therefore, we investigated whether the leucine/valine ratio in plasma could be used to diagnose patients with MSUD. Leucine/valine-ratio was calculated in controls (*n* = 900) and was 0.33–0.98 (range). In plasma samples from two patients with MSUD (classical form), the leucine/valine-ratios were 1.55 and 4.49 respectively, suggesting that this algorithm might be predictive for classical MSUD. However, more data are needed from MSUD patients to assess the value of the leucine/valine ratio as a potential diagnostic indicator for MSUD. After diagnosis, long-term management comprises a life-long strict and carefully adjusted semisynthetic diet and the ratio is therefore of less value.

Plasma of a patient with citrullinemia type I was analyzed and apart from the expected abnormal concentrations for citrulline, glutamine, alanine, arginine, and orotic acid an increased concentration (semi-quantitative) of homocitrulline was observed (Fig. 1c). The presence of homocitrulline is not only known for citrullinemia type I, but can also be found in other conditions such as hyperammonemia-hyperornithinemia-homocitrullinuria (HHH) syndrome, hyperlysinemia (Hommes et al 1983), lysinuric protein intolerance (LPI) (Kato et al 1989), and certain artificial formulas (Metwalli et al 1998). Our patient with citrullinemia type I, did not get artificial formula feeding.

Besides plasma, this method is also applicable for CSF and urine (data not shown). For the analysis of AA in CSF, adaptations in sample preparation volume and injection volume were essential. For CSF, validation studies are underway. To reduce the matrix effect, urine samples were diluted with milliQ-water, before sample preparation. Other compounds not analyzed in plasma or CSF (e.g., phospho-ethanolamine aspartylglucosamine, glycylproline, homoarginine) are currently investigated, before validation studies are initiated. Next this method for AA analysis was applied to different cell-systems (such as HEK293-cells and neuro2A-cells), cell culture supernatant, and medium. Cells were lysed in 100 % methanol and 40 μL cell-lysate or cell culture supernatant was used for AA analysis. Apart from adapting the range of the calibrators, no adaptation was needed for sample preparation or analysis of AA, suggesting that the method is robust.

Despite the advantages of the presented method, which is currently used as the front-line clinical plasma AA method in our laboratory, this method also has limitations. Currently, we are unable to separate isoleucine and D-allo-isoleucine. Several parameters including solvent concentration and pH were adapted, but did not yield satisfactory results yet (separation of isoleucine and D-allo-isoleucine). As a consequence, monitoring patients with a known diagnosis of MSUD is, for dietary compliance, thus less appropriate. Since D-allo-isoleucine is not separated from isoleucine, patients with an intermittent/variant form of MSUD will be missed. In these cases and when MSUD is suspected, plasma samples need to be analyzed with an alternative approach such as an amino acid analyzer.

Secondly, a limited set of medication and special diets were tested as interfering compounds for AA analysis. The medication and special diets examined are frequently used in the NICU settings. Results from these experiments demonstrate no interference with the AA analysis presented. However, we are aware that a limited set of medication is tested.

In conclusion a method is presented for quantification of underivatized AA in plasma by HILIC coupled to tandem mass-spectrometry. The method is fast, is easy to adapt, sensitive and reproducible, and is applicable for diagnosis and follow-up of patients with defect in amino acid metabolism.

## Electronic supplementary material

Below is the link to the electronic supplementary material.

Table 1 Absolute AA concentrations (μmol/L) of the calibration curves (DOCX 22 kb)

Table 2 Absolute concentrations of QC-low, QC-middle and QC-high (μmol/L) (DOCX 22 kb)

Table 3 AA peak areas in a random sample list. The AA peak areas of calibrator 20, 40, 60, 80 % and sample 1-24 are not depicted (DOCX 25 kb)

Table 4 Tabular representation of the solvent gradient (DOCX 20 kb)

Table 5 Slope of the calibration curve measured at three independent days (day 1, 5, and 10) (DOCX 22 kb)
