# Supplementary material for: Rapid quantification of underivatized amino acids in plasma by hydrophilic interaction liquid chromatography (HILIC) coupled with tandem mass-spectrometry
Source: J Inherit Metab Dis. 2016 Apr 21;39:651–60. doi: 10.1007/s10545-016-9935-z (PMC4987396; doi:10.1007/s10545-016-9935-z)
Supplement: Supplementary file 1 — Absolute AA concentrations (μmol/L) of the calibration curves (DOCX 22 kb) [file 10545_2016_9935_MOESM1_ESM.docx]

Table 1:

| AA | Standard 0 | Standard 1 | Standard 2 | Standard 3 | Standard 4 | Standard 5 |
| --- | --- | --- | --- | --- | --- | --- |
| Tryptophan | 0 | 50 | 100 | 150 | 200 | 250 |
| Phenylalanine | 0 | 50 | 100 | 150 | 200 | 250 |
| Leucine | 0 | 100 | 200 | 300 | 400 | 500 |
| Isoleucine | 0 | 50 | 100 | 150 | 200 | 250 |
| Valine | 0 | 100 | 200 | 300 | 400 | 500 |
| Methionine | 0 | 20 | 40 | 60 | 80 | 100 |
| Proline | 0 | 100 | 200 | 300 | 400 | 500 |
| Tyrosine | 0 | 50 | 100 | 150 | 200 | 250 |
| Pipecolic acid | 0 | 4 | 8 | 12 | 16 | 20 |
| Taurine | 0 | 50 | 100 | 150 | 200 | 250 |
| Alanine | 0 | 200 | 400 | 600 | 800 | 1000 |
| Hydroxy-proline | 0 | 10 | 20 | 30 | 40 | 50 |
| Threonine | 0 | 100 | 200 | 300 | 400 | 500 |
| Glycine | 0 | 200 | 400 | 600 | 800 | 1000 |
| Glutamine | 0 | 200 | 400 | 600 | 800 | 1000 |
| Serine | 0 | 100 | 200 | 300 | 400 | 500 |
| Asparagine | 0 | 40 | 80 | 120 | 160 | 200 |
| Citrulline | 0 | 20 | 40 | 60 | 80 | 100 |
| Glutamic acid | 0 | 50 | 100 | 150 | 200 | 250 |
| Aspartic acid | 0 | 20 | 40 | 60 | 80 | 100 |
| Histidine | 0 | 50 | 100 | 150 | 200 | 250 |
| Arginine | 0 | 50 | 100 | 150 | 200 | 250 |
| Lysine | 0 | 100 | 200 | 300 | 400 | 500 |
| Ornithine | 0 | 50 | 100 | 150 | 200 | 250 |
