# Supplementary material for: Rapid quantification of underivatized amino acids in plasma by hydrophilic interaction liquid chromatography (HILIC) coupled with tandem mass-spectrometry
Source: J Inherit Metab Dis. 2016 Apr 21;39:651–60. doi: 10.1007/s10545-016-9935-z (PMC4987396; doi:10.1007/s10545-016-9935-z)
Supplement: Supplementary file 2 — Absolute concentrations of QC-low, QC-middle and QC-high (μmol/L) (DOCX 22 kb) [file 10545_2016_9935_MOESM2_ESM.docx]

Table 2:

| AA | QC-low | QC-middle | QC-high |
| --- | --- | --- | --- |
| Tryptophan | 26 | 54 | 138 |
| Phenylalanine | 29 | 62 | 143 |
| Leucine | 64 | 129 | 193 |
| Isoleucine | 32 | 63 | 136 |
| Valine | 114 | 221 | 316 |
| Methionine | 11 | 21 | 63 |
| Proline | 137 | 247 | 264 |
| Tyrosine | 33 | 71 | 147 |
| Pipecolic acid | 0.9 | 1.8 | 6.4 |
| Taurine | 14 | 33 | 129 |
| Alanine | 181 | 357 | 624 |
| Hydroxy-proline | 7 | 10 | 30 |
| Threonine | 52 | 93 | 154 |
| Glycine | 83 | 158 | 600 |
| Glutamine | 189 | 346 | 514 |
| Serine | 43 | 98 | 173 |
| Asparagine | 14 | 28 | 65 |
| Citrulline | 13 | 26 | 64 |
| Glutamic acid | 76 | 164 | 184 |
| Aspartic acid | 4 | 11 | 32 |
| Histidine | 42 | 81 | 158 |
| Arginine | 52 | 103 | 173 |
| Lysine | 85 | 164 | 168 |
| Ornithine | 31 | 58 | 144 |
