# Supplementary material for: Rapid quantification of underivatized amino acids in plasma by hydrophilic interaction liquid chromatography (HILIC) coupled with tandem mass-spectrometry
Source: J Inherit Metab Dis. 2016 Apr 21;39:651–60. doi: 10.1007/s10545-016-9935-z (PMC4987396; doi:10.1007/s10545-016-9935-z)
Supplement: Supplementary file 3 — AA peak areas in a random sample list. The AA peak areas of calibrator 20, 40, 60, 80 % and sample 1-24 are not depicted (DOCX 25 kb) [file 10545_2016_9935_MOESM3_ESM.docx]

Table 3:

| AA | Blank | Calibrators  (20-80%) | Calibrator  (100%) | Blank | QC Low | Sample  1-24 | Sample 25 | QC High | Blank |
| --- | --- | --- | --- | --- | --- | --- | --- | --- | --- |
| Tryptophan | 0 | X | 746412 | 0 | 199516 | X | 310339 | 498736 | 0 |
| Phenylalanine | 0 | X | 940029 | 1 | 280395 | X | 363836 | 636790 | 2 |
| Leucine | 0 | X | 2676871 | 3 | 829072 | X | 1173186 | 1272945 | 25 |
| Isoleucine | 21 | X | 1576151 | 2 | 202749 | X | 288659 | 540996 | 25 |
| Valine | 0 | X | 2999426 | 0 | 516841 | X | 625808 | 729320 | 0 |
| Methionine | 0 | X | 147798 | 0 | 31535 | X | 50729 | 88994 | 0 |
| Proline | 10 | X | 4255287 | 1 | 2578387 | X | 3076637 | 2901777 | 0 |
| Tyrosine | 0 | X | 309477 | 1 | 206950 | X | 245836 | 358986 | 0 |
| Pipecolic acid | 0 | X | 531827 | 0 | 92012 | X | 124685 | 265485 | 0 |
| Taurine | 0 | X | 21650 | 0 | 2637 | X | 11190 | 10614 | 0 |
| Alanine | 0 | X | 897892 | 0 | 279602 | X | 423283 | 524482 | 0 |
| Hydroxy-proline | 0 | X | 27159 | 0 | 4229 | X | 5479 | 15933 | 0 |
| Threonine | 0 | X | 231849 | 0 | 39758 | X | 80122 | 83629 | 0 |
| Glycine | 0 | X | 57034 | 0 | 6845 | X | 14700 | 38124 | 0 |
| Glutamine | 0 | X | 681494 | 0 | 311072 | X | 380317 | 466576 | 0 |
| Serine | 0 | X | 59188 | 0 | 11864 | X | 18845 | 20437 | 0 |
| Asparagine | 0 | X | 17435 | 0 | 4246 | X | 4693 | 10298 | 0 |
| Citrulline | 0 | X | 130586 | 0 | 58792 | X | 37401 | 148792 | 0 |
| Glutamic acid | 0 | X | 87860 | 0 | 76825 | X | 30706 | 97157 | 0 |
| Aspartic acid | 0 | X | 7442 | 0 | 908 | X | 558 | 3153 | 0 |
| Histidine | 0 | X | 771054 | 0 | 706347 | X | 666332 | 1077404 | 0 |
| Arginine | 0 | X | 649484 | 0 | 492881 | X | 287004 | 741130 | 0 |
| Lysine | 0 | X | 831110 | 0 | 403606 | X | 553354 | 431582 | 0 |
| Ornithine | 0 | X | 433296 | 0 | 143799 | X | 208131 | 345007 | 0 |
