# Supplementary material for: Rapid quantification of underivatized amino acids in plasma by hydrophilic interaction liquid chromatography (HILIC) coupled with tandem mass-spectrometry
Source: J Inherit Metab Dis. 2016 Apr 21;39:651–60. doi: 10.1007/s10545-016-9935-z (PMC4987396; doi:10.1007/s10545-016-9935-z)
Supplement: Supplementary file 4 — Tabular representation of the solvent gradient (DOCX 20 kb) [file 10545_2016_9935_MOESM4_ESM.docx]

Table 4:

| Time (minutes) | Flow (mL/min) | Rate solvent A (%) | Rate solvent B (%) | Curve |
| --- | --- | --- | --- | --- |
| 0.00 | 0.400 | 100.0 | 0.0 | 0 |
| 6.00 | 0.400 | 100.0 | 0.0 | Linear (6) |
| 6.10 | 0.400 | 94.1 | 5.9 | Linear (6) |
| 10.00 | 0.400 | 82.4 | 17.6 | Linear (6) |
| 12.00 | 0.400 | 70.6 | 29.4 | Linear (6) |
| 12.10 | 0.400 | 100.0 | 0.0 | Linear (6) |
| 18.00 | 0.400 | 100.0 | 0.0 | Linear (6) |
