# Supplementary material for: Rapid quantification of underivatized amino acids in plasma by hydrophilic interaction liquid chromatography (HILIC) coupled with tandem mass-spectrometry
Source: J Inherit Metab Dis. 2016 Apr 21;39:651–60. doi: 10.1007/s10545-016-9935-z (PMC4987396; doi:10.1007/s10545-016-9935-z)
Supplement: Supplementary file 5 — Slope of the calibration curve measured at three independent days (day 1, 5, and 10) (DOCX 22 kb) [file 10545_2016_9935_MOESM5_ESM.docx]

Table 5:

| AA | Run 1 | Run 2 | Run 3 |
| --- | --- | --- | --- |
| Tryptophan | 0.0227929*x + 0.00855351 | 0.0211446*x + 0.0065324 | 0.0221359*x + 0.0149165 |
| Phenylalanine | 0.02136*x + 0.0311279 | 0.0199812*x + 0.0444618 | 0.0218651*x + 0.0615787 |
| Leucine | 0.0170227*x + 0.0520834 | 0.0178794*x + 0.00303307 | 0.0171663*x + -0.0195458 |
| Isoleucine | 0.0232637*x + 0.0622598 | 0.0229772*x + 0.0646108 | 0.0229293*x + 0.006203012 |
| Valine | 0.0120235*x + 0.178117 | 0.0123243*x + 0.146072 | 0.012177*x + 0.160534 |
| Methionine | 0.0384954*x + 0.0166802 | 0.0384287*x + 0.0729799 | 0.0396756*x + -0.00451134 |
| Proline | 0.00861726*x + 0.0251278 | 0.00829481*x + 0.0549824 | 0.00807224*x + -0.011306 |
| Tyrosine | 0.016911*x + 0.0192461 | 0.0177968*x + 0.050242 | 0.0175246*x + 0.0407275 |
| Pipecolic acid | 0.0377841*x + 0.00179993 | 0.0389202*x + 0.00652361 | 0.0395874*x + 0.00769744 |
| Taurine | 0.0179628*x + 0.189142 | 0.0183426*x + 0.201115 | 0.0175032*x + 0.129249 |
| Alanine | 0.00374768*x + 0.00860953 | 0.00386428*x + 0.0063848 | 0.00359995*x + -0.0094780 |
| Hydroxy-proline | 0.0623337*x + 0.0275803 | 0.0502246*x + 0.00667304 | 0.0681781*x + -0.0243905 |
| Threonine | 0.0338707*x + 0.0962521 | 0.0348384*x + -0.217111 | 0.0326603*x + 0.0755068 |
| Glycine | 0.00456022*x + -0.0889109 | 0.00430708*x + 0.0768151 | 0.00430401*x + -0.0541702 |
| Glutamine | 0.0101725*x + 0.254798 | 0.0108517*x + 0.219947 | 0.0110933*x + 0.265151 |
| Serine | 0.0159788*x + 0.326903 | 0.0187887*x + 0.0333593 | 0.0176761*x + 0.312433 |
| Asparagine | 0.0715494*x + 0.0325522 | 0.0769433*x + 0.100517 | 0.0822439*x + -0.184728 |
| Citrulline | 0.0643654*x + -0.034989 | 0.0575124*x + 0.0674842 | 0.0525885*x + 0.0314774 |
| Glutamic acid | 0.00958409*x + 0.0001302 | 0.00974681*x + -0.003305 | 0.0104636*x + -0.02179 |
| Aspartic acid | 0.0745358*x + -0.0354135 | 0.0675102*x + 0.0601156 | 0.0681243*x + -0.102463 |
| Histidine | 0.0130901*x + 0.0119415 | 0.0133708*x + 0.00747605 | 0.0132276*x + 0.0033822 |
| Arginine | 0.0162629*x + -0.01857 | 0.0152694*x + 0.0153379 | 0.0160296*x + -0.0144539 |
| Lysine | 0.017643*x + 0.0151922 | 0.0176668*x + 0.0903111 | 0.0179696*x + 0.0248451 |
| Ornithine | 0.0142078*x + 0.024044 | 0.0136575*x + 0.00844891 | 0.0148339*x + 0.0165475 |
